# Inhibiting Spinal Neuron-Astrocytic Activation Correlates with Synergistic Analgesia of Dexmedetomidine and Ropivacaine

**DOI:** 10.1371/journal.pone.0092374

**Published:** 2014-03-21

**Authors:** Huang-Hui Wu, Jun-Bin Yin, Ting Zhang, Yuan-Yuan Cui, Yu-Lin Dong, Guo-Zhong Chen, Wen Wang

**Affiliations:** 1 Department of Anesthesiology, Fuzhou General Hospital of Nanjing Military Region, Fuzhou, PR China; 2 Department of Anatomy, Histology and Embryology & K.K. Leung Brain Research Centre, Preclinical School of Medicine, Fourth Military Medical University, Xi’an, PR China; Zhejiang University School of Medicine, China

## Abstract

**Background:**

This study aims to identify that intrathecal (i.t.) injection of dexmedetomidine (Dex) and ropivacaine (Ropi) induces synergistic analgesia on chronic inflammatory pain and is accompanied with corresponding “neuron-astrocytic” alterations.

**Methods:**

Male, adult Sprague-Dawley rats were randomly divided into sham, control and i.t. medication groups. The analgesia profiles of i.t. Dex, Ropi, and their combination detected by Hargreaves heat test were investigated on the subcutaneous (s.c.) injection of complete Freund adjuvant (CFA) induced chronic pain in rat and their synergistic analgesia was confirmed by using isobolographic analysis. During consecutive daily administration, pain behavior was daily recorded, and immunohistochemical staining was applied to investigate the number of Fos-immunoreactive (Fos-ir) neurons on hour 2 and day 1, 3 and 7, and the expression of glial fibrillary acidic protein (GFAP) within the spinal dorsal horn (SDH) on day 1, 3, 5 and 7 after s.c. injection of CFA, respectively, and then Western blot to examine spinal GFAP and β-actin levels on day 3 and 7.

**Results:**

i.t. Dex or Ropi displayed a short-term analgesia in a dose-dependent manner, and consecutive daily administrations of their combination showed synergistic analgesia and remarkably down-regulated neuronal and astrocytic activations indicated by decreases in the number of Fos-ir neurons and the GFAP expression within the SDH, respectively.

**Conclusion:**

i.t. co-delivery of Dex and Ropi shows synergistic analgesia on the chronic inflammatory pain, in which spinal “neuron-astrocytic activation” mechanism may play an important role.

## Introduction

Analgesia with local anesthetics (LAs) has been proved to be effective in neuraxial block [1]–[5]. However, the catastrophic neural complications [6] and great impacts on motor function restrict the intrathecal (i.t.) application [7], [8]. Ropivacaine (Ropi) achieves less impact on motor function in a low concentration and favorable safety profile compared with other LAs. However, repeated i.t. Ropi also dose-dependently induces neurotoxicity and triggers neuronal apoptosis [9]–[11]. Thus, physicians tend to prefer the “combination analgesia” with low dose of Ropi and another adjuvant that employs different but somehow overlapped mechanism, may lead to the synergistic analgesia, *via* enhancing the shared pathways or complementing the independent pathways.

Dexmedetomidine (Dex), a highly selective α_2_-adrenergic receptors (α_2_AR) agonist, exhibits analgesia when systemically administered [12]. Besides, perineural Dex also facilitates the analgesia of Ropi in peripheral nerve block [13], [14]. However, there is no experimental evidence concerning the synergistic analgesia of i.t. co-delivery of Dex and Ropi, and also far from being revealed about the underlying mechanisms for their synergism.

The current evidences inspired us that co-delivery of Ropi and Dex might induce synergistic analgesia *via* inhibiting the activity of neurons and astrocytes. First, the analgesic mechanisms for Ropi include blocking fast voltage-gated sodium channels on neuronal axons [15], and suppressing glial activations [15] within the spinal dorsal horn (SDH), etc. Second, studies of others [16]–[18] and ours [19]–[24] suggest that crosstalk between neurons and astrocytes contributes to the initiation and maintenance of pain. Third, α_2_ARs are expressed on not only neurons but also astrocytes [25], [26]. Activation of α_2_AR not only reduces noxious stimuli evoked release of nociceptive substances [27], [28] from the primary afferent fibers, but also inhibits the spinal astrocytic activation.

Hence, we designed the current experiment to test the hypothesis that the i.t. co-delivery of Dex and Ropi at lower dosages yields synergistic analgesia and the underlying mechanism correlates with inhibition on the spinal neuron-astrocytic activation.

## Materials and Methods

All experimental procedures received prior approval from the Animal Use and Care Committee for Research and Education of the Fourth Military Medical University (Xi’an, China), and enacted according to the guidelines of the International Association for the Study of Pain [29]. All efforts were made to minimize animal suffering and to reduce the number of animals used.

### Animal preparation

Male *Sprague-Dawley* (SD) rats (180–220 g) provided by Experimental Animal Center of the Fourth Military Medical University were housed in standard transparent plastic cages with a 12/12 h light/dark cycle (light on at 08:00 am) under 22–25°C ambient temperature with food and water available. Before experiments, animals were allowed to habituate to the housing environment for 7 d.

### Drugs

Dex (Precedex, 200 μg/2 ml ), lidocaine (Lido, 100 mg/5 ml) and Ropi (Naropin, 100 mg/10 ml) were purchased from Nhwa Pharmaceutical Co., Ltd. (Jiangsu, China), Hualu Pharmaceutical Co., Ltd. (Shandong, China), and AstraZeneca AB. (Sweden), respectively. CFA (10 mg/10 ml) was purchased from Sigma-Aldrich Co. LLC.. Artificial cerebral spinal fluid (ACSF: NaCl 124 mM, D-Glucose 10 mM, NaH_2_PO_4_ 1 mM, NaHCO_3_ 25 mM, MgSO_4_ 1 mM, KCl 4.4 mM and CaCl_2_·H_2_O 2 mM) was used as vehicle for the i.t. injection and diluting Ropi (100 mg/10 ml) to the target concentrations (1 mg/ml). Dex was used in its original concentration (200 μg/2 ml).

### Experimental protocols

**Figure 1:**
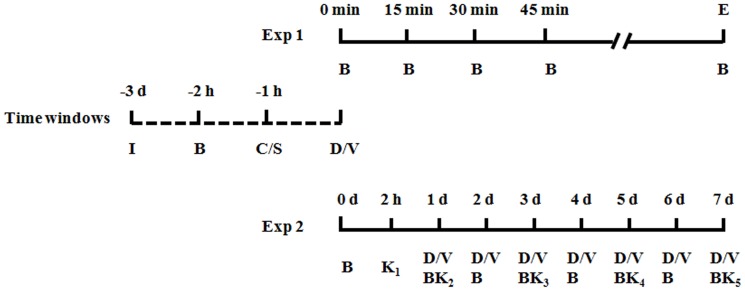
Experimental protocol. Time window indicated the days after s.c. CFA or saline injection. I: Intrathecal catheter implantation; B: Behavior test; C: CFA subcutaneous injection; S: Saline subcutaneous injection ; D: Drugs intrathecal injection; V: Vehicle intrathecal injection; E: End of the effect of i.t. medication; K: Killing the rats for further immunofluorescence histochemical staining, western blot, and HE staining.

Experimental protocols were summarized in **Figure 1**. The basal paw withdrawal latencies (PWLs) to noxious thermal stimuli were calculated from the measurements at 1 h prior to i.t. intubation and subcutaneous (s.c.) CFA injection, respectively.

Experiment 1 was designed to evaluate the analgesia profiles of i.t. delivery of Dex, Ropi, or their combination (based on the theoretical 50% effective dose (ED_50_) for their combination (ED_50add_)) and identify whether there is synergistic analgesia according to the actual ED_50_ for combination (ED_50comb_) by isobolographic analysis. In this section, rats were randomly assigned to one of the following groups: (1) rats receiving i.t. injection with 10 μl of ACSF followed by s.c. injection with 100 μl of saline 1 h later (Saline-Veh group); (2) rats receiving i.t. injection with 10 μl of ACSF followed by s.c. injection with 100 μl of CFA 1 h later (CFA-Veh group); (3) rats receiving different dose regimes of i.t. Dex, Ropi and their combination injection (Dex: 0.5, 1, and 2 μg/200 g; Ropi: 5, 10, and 20 μg/200 g; Dex&Ropi: ED_50add*1/10_, ED_50add*2/10_, ED_50add*4/10_ and ED_50add*8/10_) followed by s.c. injection with 100 μl of CFA 1 h later. PWLs to noxious thermal stimuli were measured a 15 min interval until analgesic effect faded away.

Experiment 2 was designed to evaluate the effect of i.t. delivery of ED_50_ Dex, ED_50_ Ropi, or ED_50comb_ Dex&Ropi on pain behaviors and neuron-astrocytic activations. In this section, i.t. delivery of medication or vehicle was made 30 min after s.c. CFA/saline injection. Then, from day 0 to 7, daily pain behavior was tested at 30 min after daily i.t. medication. Rats in this section were sacrificed at 2 h, 1, 3, and 7 d after s.c. CFA injection for Fos immunofluorescence histochemical staining, at 1, 3, 5 and 7 d for glial fibrillary acidic protein (GFAP) staining, and at 3 and 7 d for Western blot.

Supplemental experiments were designed to identify whether repeated i.t. Dex&Ropi caused potential tachyphylaxis or sensitization by investigating the analgesic duration and intensity after repeated i.t. ED_50comb_ Dex&Ropi. Open field (OF) and rotarod tests on naïve rats free from CFA injection or pain behavioral observation after maximal dosages of medications (or their combination) delivery were performed to exclude the potential influence on intubation procedure and i.t. medication that might influence motor function and bias our behavioral readout. Besides, pathology on SDH were examined by hematoxylin-eosin (HE) staining (details were presented in **Supporting Information**).

The dosages of i.t. Dex and Ropi applied in the present study were based on the previous reports [30], [31] and our pilot experiment. All behavioral tests were performed during 8–12 am and conducted by an observer blinded to the experimental condition.

### CFA-induced chronic inflammatory pain model

Firstly, i.t. intubation was performed under 2% (w/v) sodium pentobarbital anesthesia (45 mg/kg, i.p.) according to our previous studies [19]–[21]. Briefly, a midline incision (3 cm) was cut on back of the rat at the level of the thoracic vertebrae. A pre-measured PE-10 tube (ID 0.28 mm and OD 0.61 mm) was passed caudally from T_8_ to L_3_ level of the spinal cord, fixed at the back of rat’s ears through s.c. tunnel, with 2 cm free end exposed in the upper thoracic region. Only the rats judged as no neurological deficit and presented complete tail and bilateral hind legs paralysis after i.t. Lido (2%, 10 μl) were used in the followed experiments.

After 3 d recovery, CFA-induced pain model was established according to our previous study [32]. Briefly, rats were re-anesthetized with 2% isoflurane in O_2_, and received s.c. injection with 100 μl of CFA (1 mg/ml; Sigma-Aldrich, St. Louis, MO, USA) or 0.9% normal saline into the plantar side of the left hind paw.

### Hargreaves test

Thermal hyperalgesia was investigated using Hargreaves method [33]. Paw withdrawal in response to noxious thermal stimuli was assessed using an RTY-3 radiant heat stimulator (Xi’an Fenglan Instrumental Factory, Xi’an, China). Rats were placed in plastic boxes on a glass plate for at least 30 min before testing. The time from initiation of the light beam to paw withdrawal was recorded as PWL. The intensity of the beam was set to produce a basal PWL of approximate 14–16 s. A cut-off time of 35 s was set to prevent excessive tissue damage due to repeated application of the thermal stimuli.

### Immunofluorescence histochemical staining

Rats were deeply anesthetized with sodium pentobarbital (65 mg/kg, i.p.) and perfused through the ascending aorta with 100 ml of 0.9% normal saline followed by 500 ml of 0.1 M phosphate buffer (PB, pH 7.2–7.4) containing 4% (w/v) paraformaldehyde and 30% (v/v) picric acid. After perfusion, the L_4-5_ spinal segments were removed and postfixed in the same fixative for 2–4 h and then cryoprotected for 48 h at 4°C in 0.1 M PB that contained 30% (w/v) sucrose. After being embedded in an inert mounting medium (OCT; Tissue-Tek; Sakura; Torrance, CA, USA), transverse frozen spinal sections (30 μm thick) were cut in a cryostat (CM1800; Leica, Heidelberg, Germany) and collected serially. The sections were rinsed in 0.01 M phosphate-buffered saline (PBS, pH 7.2–7.4) 3 times (10 min each), blocked with 10% fetal calf serum (FCS) in 0.01 M PBS containing 0.3% (v/v) Triton X-100 for 60 min at room temperature (RT), followed by immunofluorescent labeling.

The sections were incubated overnight at 4°C with one of the following primary antibodies: mouse anti-GFAP antibody (1∶4000; Chemicon, Temecula, CA, USA) and rabbit anti-Fos antibody (1∶1000; Santa Cruz Biotechnology, Santa Cruz, CA, USA), respectively, in the antibody dilution medium. The medium consisted of 0.01 M PBS (pH 7.4) containing 5% (v/v) normal donkey serum (PBS-NDS), 0.3% (v/v) Triton X-100, 0.05% (w/v) NaN_3_ and 0.25% (w/v) λ-carrageenan. The sections were washed three times with 0.01 M PBS (10 min each) and then incubated for 4 h at 22–25°C with the secondary antibody: Alexa 488 donkey anti-mouse and Alexa 594 donkey anti-rabbit IgG (1∶500; Amer Control Pharmacia Biotech Inc., Piscataway, NJ, USA), respectively, diluted in the antibody dilution medium. The specificity of the staining was tested on the sections from another dish by omitting the specific primary antibody. Finally, the sections were obtained using a confocal laser scan microscope (FV1000; Olympus, Tokyo, Japan; 1 μm thick optical section).

### Western blot

Rats were sacrificed under deep anesthesia (2% (w/v) sodium pentobarbital, 65 mg/kg, i.p.) and the L_4-5_ SDHs were quickly removed and then dissected using the “open book” method [34]. Briefly, the spinal segment was cut into a left and right half from the midline, and the left half was further split into the dorsal and ventral horns at the level of the central canal. The selected region was homogenized with a hand-hold pestle in sodium dodecyl sulfate (SDS) sample buffer (60 μl/mg tissue) containing proteinase inhibitors. The electrophoresis samples were heated at 100°C for 5 min and loaded onto 10% SDS-polyacrylamide gels with standard Laemmli solutions (Bio-Rad Laboratories, CA, USA). After the proteins were electroblotted onto a polyvinylidene difluoride membrane (PVDF, Immobilon-P, Millipore, Billerica, MA, USA), the membranes were placed in a blocking solution containing Tris-buffered saline with 0.02% Tween (TBS-T) and 5% non-fat dry milk, and incubated 60 min under gentle agitation at RT, then 4°C for overnight with mouse anti-GFAP antibody (1∶4000; Chemicon, Temecula, CA, USA), or mouse anti-β-actin antibody (1∶5000; Sigma, St Louis, MO, USA), respectively. Bound primary antibodies were detected by incubation with anti-mouse horseradish peroxidase-conjugated secondary antibody (1∶10000; Amersham Pharmacia Biotech Inc., Piscataway, NJ, USA) for 2 h under gentle agitation at RT. Between each step, the immunoblots were rinsed with TBS-T for 3 times (10 min each). Protein blots’ densities were detected and analyzed in the Bio-Rad ChemiDoc Imaging System (Bio-Rad Laboratories Ltd, USA).

### Dose-effect curve and ED_50_ calculation

The dosages of i.t. Dex, Ropi, and their combination were transformed into logarithm dose and the non-line fit was performed so as to build the dose-effect curve. Based on the dose-effect cure, the ED_50_s of each agent on analgesia was calculated.

### Isobolographic analysis

An isobolographic analysis was further performed to characterize drug interaction according to the method originally described by Tallarida [35] and in our previous report [36]. Both drugs in Experiment 1 achieved comparable levels of anti-nociception so that ED_50_ values were used to obtain a theoretical dose-response curve for a fixed-ratio combination of Dex and Ropi [35], [36].

We calculated a theoretical ED_50add_ based on the theoretical dose-response curve. Subsequently, an experimental dose response curve was obtained by treating animals with one of the following combination doses: ED_50add*1/10_, ED_50add*2/10_, ED_50add*4/10_ and ED_50add*8/10_ in a fixed-ratio of 1∶1 for Dex and Ropi. According to this dose-response curve, the ED_50_ of combination could be calculated and presented as ED_50comb_. An ED_50comb_ less than ED_50add_ suggested a synergistic effect of these two agents.

### Statistical analysis

Data of normal distribution and homogeneous variance were expressed as mean ± standard error mean (SEM), and analyzed by researchers blinded to the surgery and reagents used. One- or two-way analysis of variance (ANOVA) followed by *Bonferroni’s post hoc* test was used for multiple comparison. The area under the time–course curves (AUCs) values during the analysis time was used to measure the summed effects of different drugs as described in our previous studies [36], [37]. Besides, *Kruskal-Wallis* test with *Dunn's* multiple comparison test was performed to analyzed the data of inhomogeneous variance. All these data were analyzed by using GraphPad Prism version 5.01 for Windows (Graph Pad Software, San Diego California USA, www.graphpad.com). *P*<0.05 was considered as statistical significance.

## Results

### Effect of i.t. Dex on CFA-induced hyperalgesia

Consistent with previous studies [38], [39], CFA injection produced long-term thermal hyperalgesia as evidenced by significant decreases in PWLs, indicating that a successfully induced chronic inflammatory pain by s.c. CFA.

**Figure 2:**
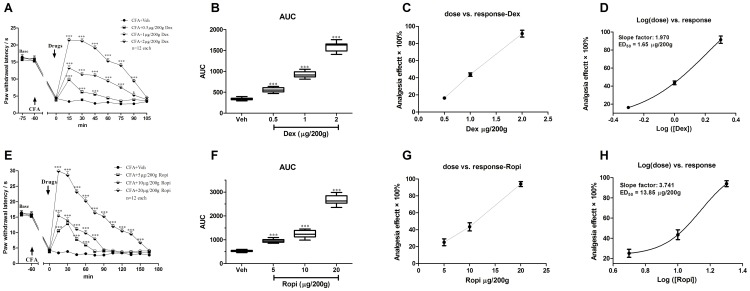
i.t. Dex or Ropi dose-dependently inhibited CFA-induced thermal hyperalgesia of the injected hind paw. Analgesia duration of different doses of i.t. Dex or Ropi was shown in **A** and **E**. The AUCs for different groups were calculated to perform statistical analysis (**B**) and (**F**). The dose-effect or log (dose)-effect curves for the analgesic effects in attenuating CFA-induced thermal hyperalgesia after i.t. vehicle and Dex or Ropi were shown in **C** or **G** and **D** or **H**, respectively.* *p*<0.05; *** *p*<0.001, compared with CFA-Veh group; arrows indicated s.c. CFA injection and i.t. intervention time point, respectively.

Compared with CFA-Veh group, i.t. Dex significantly elevated PWLs in a dose-dependent manner (**Figure 2A**; two-way ANOVA, *P*<0.001). As summarized in AUC values of PWLs, the analgesia of i.t. Dex presented a significant group difference among 3 dose regimes (**Figure 2B**; one-way ANOVA, *P* = 0.0055). The average valid analgesic duration was dose-dependently prolonged (0.5 *vs.* 1 *vs.* 2 μg/200 g: 45 *vs.* 90 *vs.* 90 min). *Bonferroni’s post hoc* test also revealed group difference between 0.5, 1, or 2 μg/200 g and vehicle groups (*P*<0.001). Besides, the effects of different doses of i.t. Dex on the PWLs were calculated based on the log (dose) *vs.* response curve (**Figure 2D**) from the dose *vs.* response curve (**Figure 2C**). ED_50_ of i.t. Dex analgesia was 1.65 μg/200 g.

### Effect of i.t. Ropi on CFA-induced hyperalgesia

Compared with CFA-Veh group, i.t. Ropi also significantly elevated PWLs in a dose-dependent manner (**Figure 2E**; two-way ANOVA, *P*<0.001). As summarized in AUC values of PWLs, the analgesia effects of i.t. Ropi presented a significant group difference among 3 dose regimes (**Figure 2F**; one-way ANOVA, *P* = 0.0001). The averaged valid analgesic duration was dose-dependently prolonged (5 *vs.* 10 *vs.* 20 μg/200 g: 60 *vs.* 75 *vs.* 150 min). *Bonferroni’s post hoc* test also revealed group difference between 5, 10, or 20 μg/200 g and vehicle groups (*P*<0.001). Besides, the effects of different doses of i.t. Ropi on the PWLs were calculated based on the log (dose) *vs.* response curve (**Figure 2H**) from the dose *vs.* response curve (**Figure 2G**). The ED_50_ of i.t. Ropi analgesia was 13.85 μg/200 g.

### Effect of i.t. Dex and Ropi combination on CFA-induced hyperalgesia

**Figure 3:**
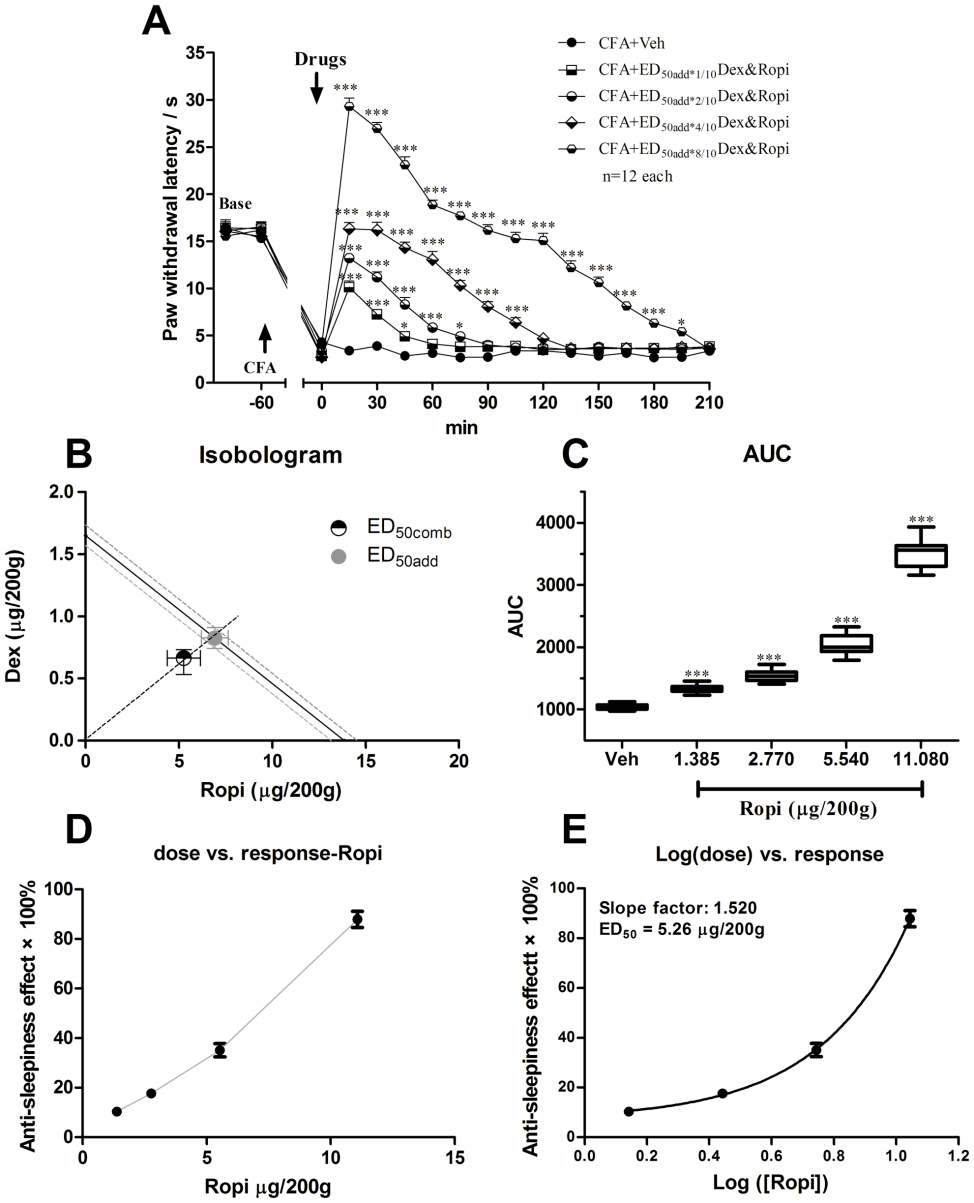
i.t. Dex and Ropi combinations dose-dependently inhibited CFA-induced thermal hyperalgesia of the injected hind paw. i.t. Dex and Ropi combinations dose-dependently prolonged analgesia duration (**A**). Iosobologram for combination analgesia was shown in **B**. The AUCs for different groups were calculated to perform statistical analysis (**C**). The dose-effect or log (dose)-effect curves for combination analgesic effects were shown in **D** and **E**. * *p*<0.05; *** *p*<0.001, compared with CFA-Veh group; arrows indicated s.c. CFA injection and i.t. intervention time point, respectively.

Due to the different analgesic profile of i.t. Dex and Ropi, interaction parameters were calculated on the basis of the anti-nociceptive effects exerted by two drugs. Middle dose response curves of both compounds were linear, thus, a composite additive curve was constructed (**Figure 3A**). Additive regression allowed us to calculate theoretical ED_50_ for a fixed-ratio (1:1) combination of Dex and Ropi (ED_50add_ =  0.83 Dex + 6.93 Ropi). The dose regime designed to investigate the experimental ED_50comb_ included the following combinations: 0.17 Dex + 1.39 Ropi (ED_50add*1/10_ Dex&Ropi), 0.33 Dex + 2.77 Ropi (ED_50add*2/10_ Dex&Ropi), 0.66 Dex + 5.54 Ropi (ED_50add*4/10_ Dex&Ropi) and 1.32 Dex + 11.08 Ropi (ED_50add*8/10_ Dex&Ropi).

i.t. Dex and Ropi combination significantly elevated PWLs in a dose-dependent manner (**Figure 3A**; two-way ANOVA, *P*<0.001). As summarized in AUC values of PWLs, the analgesia effects of i.t. Dex and Ropi combination presented a significant group difference among 4 dose regimes (**Figure 3C**; one-way ANOVA, *P*<0.001). The averaged valid analgesic duration was dose-dependently prolonged (Dex&Ropi ED_50add*1/10_ *vs.* ED_50add*2/10_ *vs.* ED_50add*4/10_ *vs.* ED_50add*8/10_: 45 *vs.* 75 *vs.* 105 *vs.* 195 min). *Bonferroni’s post hoc* test also revealed group difference between Dex&Ropi ED_50add*1/10_, ED_50add*2/10_, ED_50add*4/10_ or ED_50add*8/10_ and vehicle groups (*P*<0.001). The experimental ED_50comb_ calculated from these dose-response curves (**Figure 3D and 3E**) for pain responses was 0.63 Dex + 5.26 Ropi. Isobolographic analysis of Dex and Ropi concomitant effect in nociceptive test showed the ED_50comb_ was smaller than the lower (95%) range of ED_50add_, suggesting that the interaction between the two drugs was synergistic (**Figure 3B**). These results revealed that the synergistic analgesia facilitated to prolong analgesic duration and to enhance analgesic intensity with less dose of Dex and Ropi.

### Effect of i.t. Dex as an adjuvant on CFA-induced chronic hyperalgesia

We have confirmed a synergistic effect of i.t. delivery of Dex and Ropi combination in a short-term observation. Then, the effect of i.t. Dex as an adjuvant on CFA induced chronic hyperalgesia was further explored.

According to Experiment 1, ED_50_ Dex, ED_50_ Ropi, or ED_50comb_ Dex&Ropi were administered to achieve equal 50% analgesia, the dose regime designed in this section were as the following: 1.65 Dex (CFA-Dex), 13.85 Ropi (CFA-Ropi), and 0.63 Dex + 5.26 Ropi (CFA-Dex&Ropi).

**Figure 4:**
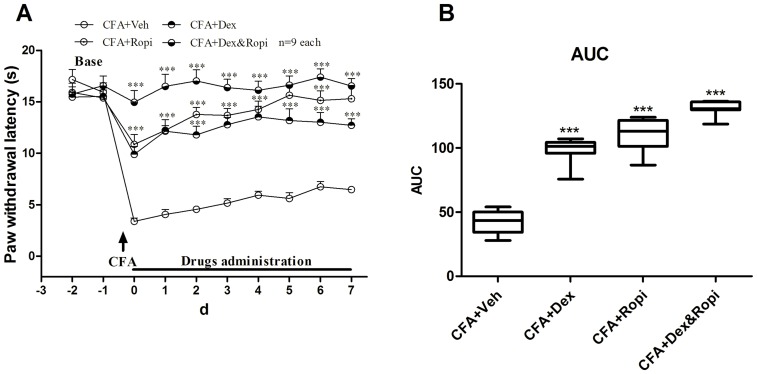
Effect of i.t. Dex, Ropi, or their combination on CFA-induced chronic hyperalgesia. Analgesia proporties of i.t. delivery of individual and concomitant medications during 7 d after s.c. CFA injection were shown in **A**. The AUCs for different groups were calculated to perform statistical analysis (**B**). *** *p*<0.001, compared with CFA+Veh group; arrows indicated s.c. CFA injection time point.

Anti-nociceptive effects were observed in all intervention groups (**Figure 4A**; two-way ANOVA, *P*<0.001) from day 0 to 7. As summarized in AUC values of PWLs, the analgesia effects presented a significant group difference among 3 intervention strategies (**Figure 4B**; one-way ANOVA, *P*<0.001). *Bonferroni’s post hoc* test also revealed group difference between CFA-Dex, CFA-Ropi, or CFA-Dex&Ropi and CFA-Veh group (*P*<0.001). Meanwhile, there was significant difference between CFA-Dex (*P*<0.001) or CFA-Ropi (*P*<0.01) and CFA-Dex&Ropi groups, while no significant difference between CFA-Dex and CFA-Ropi group (*P* > 0.05), suggesting that a synergistic effect might also be beneficial in a long-term treatment because although the dose of ED_50comb_ Dex&Ropi was lower than individural ED_50_, better analgesic effects were achieved.

### Effect of i.t. Dex, Ropi or their combination on CFA-induced neuronal activation

Since the initiation of chronic pain predominantly correlates with the neuronal activation, we wondered whether the synergistic analgesia was due to the facilitated neuronal inhibition when ED_50_ Dex, ED_50_ Ropi, or ED_50comb_ Dex&Ropi was administrated to achieve 50% analgesia.

**Figure 5:**
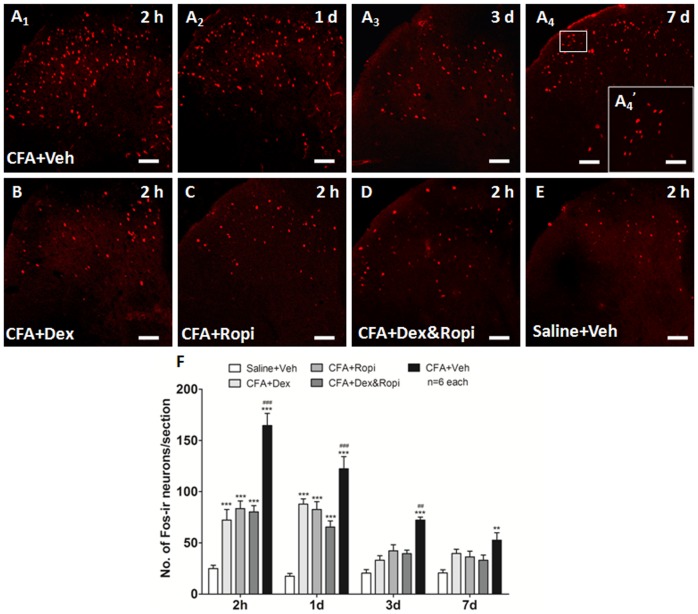
Effect of i.t. Dex, Ropi, or their combination on CFA-induced neuronal activation. s.c. CFA-induced thermal hyperalgesia was accompanied with the increase in the number of Fos-ir neurons to the peak at 2 h after injection, and gradually reduced but sustained to day 7 in the ipsilateral SDH (**A_1_∼A_4_**). Both i.t. individual and concomitant medications significantly inhibited neuronal activation indicated by less number of Fos-ir neurons in the ipsilateral SDH at 2 h after s.c. CFA injection (**B∼E**). Number of Fos-ir neurons was presented in **F** at 2 h, 1, 3 and 7 d after s.c. CFA injection, respectively. * p<0.05, ** *p*<0.01, *** *p*<0.001, compared with Saline-Veh group; ## *p*<0.01, ### *p*<0.001, compared with CFA-Dex&Ropi group. Scalebars = 10 μm in **A_4_^’^**, 50 μm in **A_1_∼A_4_** and **B∼E**.

We observed that significantly increased neuronal activation indicated by the nuclei expression of Fos and such neuronal activation reached the peak at 2 h after s.c. CFA injection (CFA-Veh 2 h *vs.* 1 d *vs.* 3 d *vs.* 7 d = 164.67±11.86 *vs.* 122.33±11.94 *vs.* 72.33±2.91 *vs.* 52.67±7.26, one-way ANOVA with *Bonferroni’s post hoc* test, *P*<0.001). These Fos-ir neurons predominantly located in the superficial layers of the L_5_ SDH in vehicle treated rats. Meanwhile, the number of Fos-ir neurons also presented a temporal change that reached its peak at 2 h and gradually reduced but sustained to day 7 (**Figure 5A** **_1_∼A_4_**). Considering 2 h after s.c. CFA injection as the peak of neuronal activation, photomicrographs of Fos-ir neurons in the ipsilateral L_5_ SDH in each group at this time-point were shown in **Figure 5B** **∼E**. The total number of Fos-ir neurons/section in these groups was shown in **Figure 5F**. There was a significant group difference in the numbers of spinal Fos-ir neurons at 2 h after s.c. CFA injection (**Figure 5F**; one-way ANOVA, *P*<0.001). *Bonferroni’s post hoc* test also revealed a significant group difference between CFA-Dex, CFA-Ropi, or CFA-Dex&Ropi and Saline-Veh or CFA-Veh group (*P*<0.001), respectively, at 2 h. The number of Fos-ir neurons reduced to the equal with Saline-Veh group at day 3 (*P* > 0.05). However, no significant difference was observed among CFA-Dex, CFA-Ropi, and CFA-Dex&Ropi (*P* > 0.05). These results suggested that a synergistic effect might exist because although the dose of ED_50comb_ Dex&Ropi was lower than individural ED_50_, equal inhibition on the neuronal activation in the initiation phase was achieved.

### Effect of i.t. Dex, Ropi or their combination on CFA-induced astrocytic activation

It seems that synergistic inhibition of the neuronal activation contributes to the initiation of the chronic pain, however, what contributes to the long-term synergistic analgesia of Dex and Ropi? Our previous studies [19]–[21], [23], [24] suggested that the maintenance of chronic pain correlated with the astrocytic activation. Thus, we further wondered that whether the astrocytic inhibition contributed the superiorities to synergistic analgesia when ED_50_ Dex, ED_50_ Ropi, or ED_50comb_ Dex&Ropi was administrated to achieve equal 50% analgesia.

**Figure 6:**
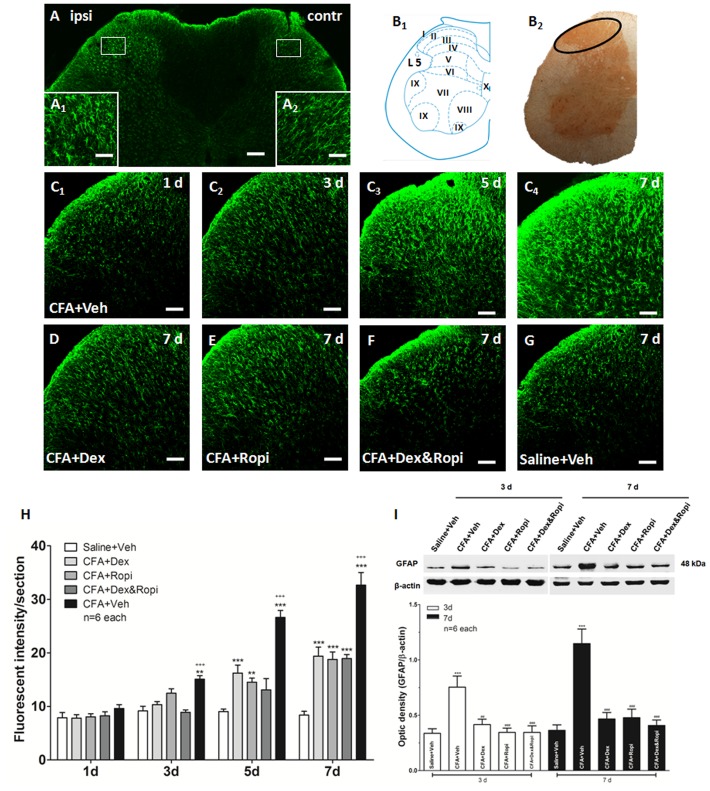
Effect of i.t. Dex, Ropi, or their combination on CFA-induced astrocytic activation. s.c. CFA injection induced appreciable astrocytic activation and GFAP up-regulation in the ipsilateral SDH (**A**). The activated astrocytes presented hypertrophied cell bodies and the thickened processes (**A_1_**), compared with contralateral side (**A_2_**). Scheme showed an overview of detected region (laminae I∼III) of immunohistochemical quantification and western blot (**B_1_** and **B_2_**). CFA-induced aroused GFAP activation from day 3, and reached the peak on day 7 in the ipsilateral SDH (**C_1_∼C_4_**). i.t. individual or concomitant medication down-regulated GFAP expression in the ipsilateral SDH on day 7 (**D∼G**). Fluorescent intensities of GFAP expression at 1, 3, 5 and 7 d after s.c. CFA inejction were presented in **H**. Western blot of GFAP expression at 3 and 7 d after s.c. CFA injection was shown in **I**. ** *p*<0.01, *** *p*<0.001, compared with Saline-Veh group; ## *p*<0.01, ### *p*<0.001, compared with CFA-Veh group; +++ *p*<0.001, compared with CFA-Dex&Ropi group. Scale bars = 100 μm in **A**, 10 μm in **A_1_** and **A_2_**, 50 μm in **C_1_∼C_4_** and **D∼G**.

Firstly, we observed that CFA aroused a significant astrocytic activation indicated by GFAP up-regulation in the ipsilateral compared with the contralateral SDH with a temporal change (CFA-Veh) (**Figure 6A**). Immunohistochemistry indicated that activated astrocytes presented as hypertrophied cell bodies and thickened processes with enhanced GFAP-ir (**Figure 6A** **_1_** and **A_2_**). Scheme showed an overview of detected region (laminae I∼III) for immunohistochemical quantification and Western blot (**Figure 6B** **_1_** and **B_2_**). GFAP up-regulation was not obvious on day 1, but significant on day 3 and reached a peak on day 7 (**Figure 6C** **_1_**∼**C_4_** and **6H**). This finding was consistent with our previous publication [24] that astrocytic activation remained at high levels at 3 weeks after spinal nerve ligation (SNL). This increase characteristics was also confirmed in our Western blot that peak GFAP expressions on day 7 after s.c. CFA injection were 0.363±0.050 in Saline-Veh compared with 1.147±0.132 in CFA-Veh group (*P*<0.001) (**Figure 6I**).

Then, we administered i.t. ED_50_ Dex, ED_50_ Ropi, or ED_50comb_ Dex&Ropi once daily from day 0 to 7 to observe the effects on the CFA induced astrocytic activation. Consistent with our previous study [19] on a neuropathic pain model, we observed that astrocytic activation reached its peak with abundant GFAP expression on day 7 (**Figure 6D**∼**G**). There was a significant group difference in the GFAP expression (**Figure 6H**; one-way ANOVA, *P*<0.001). However, *Bonferroni’s post hoc* test revealed that no group difference between CFA-Dex or CFA-Ropi and CFA-Dex&Ropi groups (*P* > 0.05). Interestingly, an appreciably low expression tendency in CFA-Dex&Ropi group was observed when astrocytes were initially activated on day 3, suggesting that i.t. concomitant strategy might reduce astrocytic activation at early stage, although no significant difference was observed among intervention groups.

Western blot showed that GFAP in all intervention groups were significantly decreased on day 3 and 7. Similarly, there was no significant difference among intervention groups between CFA-Dex or CFA-Ropi and CFA-Dex&Ropi groups (*P* > 0.05). (**Figure 6I**). These results suggested that a synergistic effect might also be beneficial in a long-term treatment because although the dose of ED_50comb_ Dex&Ropi was lower than individural ED_50_, equal effect of astrocytic inhibition in the maintenance phase was achieved.

### Effect of repeated i.t. co-delivery of Dex and Ropi

Results from this supplemental experiment suggested that repeated i.t. Dex and Ropi combination presented a stable analgesia property without any acute tachyphylaxis or sensitization (details were presented in **Figure S1**).

### Effect of i.t. medications on motor function

Results from this supplemental experiment suggested that the intubation procedure and i.t. medication didn’t impair rats’ locomotion and motor coordination function indicated by OF and rotarod test, respectively (details were presented in **Figure S2**).

### Effect of i.t. medications on pathology

Results from this supplemental experiment suggested that i.t. Dex didn’t cause any pathological change in SDH, instead, it tended to attenuate Ropi-induced acute neuroinflammation at 24 h after CFA injections (details were presented in **Figure S3**).

## Discussion

To our knowledge, this is the first report discussing a synergistic analgesia for i.t. Dex and Ropi in a CFA-induced rat chronic pain model. Our data supported that i.t. Dex and Ropi combination in a fixed ratio yielded synergism, and the possible mechanism correlated with neuron-astrocytic interaction. Less Fos-ir neurons in pain “initiation phase” as well as remarkably decreased astrocytic activation in “maintenance phase” indicated the overall pain control by i.t. concomitant strategy.

### i.t. Dex, Ropi or their combination reduces thermal hyperalgesia via inhibiting neuronal and astrocytic activation in the spinal cord

According to previous studies [17] and our experimental data, spinal neuronal activation might be dominant in pain “initiation phase” presented as obvious thermal hyperalgesia with increased Fos-ir neurons within 3 d, particularly in the first 24 h after s.c. CFA injection. On the contrary, astrocytes might be critical in pain “maintenance phase”, since spinal Fos-ir neurons have been down-regulated after “acute phase”, but GFAP up-regulated in “chronic phase” of pain [24], [40], [41], which presented sustained thermal hyperalgesia to 7 d after s.c. CFA injection. Astrocytes express receptors for various neurotransmitters, which enable them to respond to neural signals and thus be activated [40], [42]–[45]. Activated astrocytes produce numerous mediators such as pro-inflammatory cytokines and growth factors that enhance neuronal activity [46], [47]. Using specific metabolic inhibitors for astrocytes such as fluorocitrate, studies have indicated that astrocytic activation is required and sufficient for chronic pain sensitization [48].

Based on previous studies and our experimental data, we hypothesized that the synergistic analgesic mechanisms of i.t. Dex and Ropi might be due to i) both Dex and Ropi block nociceptive stimuli to transmit, leading to the directly inhibition on neuronal activation *via* sodium channels or/and α_2_ARs located on neurons membranes in pain “initiative phase”; ii) Dex might directly inhibit astrocytic activation, and then, interrupt the neuron-astrocyte cross-talk in pain “maintenance phase”; iii) Ropi indirectly inhibits asctrocytic activation deriving from blocking neuronal sensitization or directly suppresses spinal asctrocytic activation in a nerve growth factor (NGF)-independent manner [15], and then, achieves in subsequent synergistic analgesia by interrupting the neuron-astrocytic cross-talk. However, the specific molecular mechanisms require further discussion.

In the current study, we didn’t investigate the time-course and the effects of Dex and Ropi combination on microglia, since our previous study [22] suggested that the peak activation of microglia was at day 3 in a neuropathic pain model, and thus it might be predominant in “sub-acute phase” instead of “chronic phase”. However, as a critical component of the “cross-talk” between neuron-glia, microglia also participates in the pain cascade. It needs our further work to identify whether microglia plays an important role in pathological pain, as well as the inhibition of microglia contributes to the facilitations of i.t. Dex and Ropi combination on improving chronic inflammatory pain.

### i.t. Dex as an adjuvant may be a new analgesic strategy

Previous studies [49], [50] have indicated that LAs had significant neurotoxicity both in *vivo* and in *vitro*. Our results demonstrated that i.t. Dex tended to attenuate Ropi-induced acute neuroinflammation at 24 h after s.c. CFA injection (**Supporting Information**). Recent studies have also confirmed the neuroprotective or growth-promoting properties of Dex in many tissues, such as protecting neurons after stroke by promoting glial cell line-derived neurotrophic factor (GDNF) release [51], protected cortex neurons from apoptosis [52], [53], and decreased Lido-induced cortical neuron death indicated by the decreased expressions of caspase-3 [31].

Although the half-life time of Dex is short (2∼3 h), its anxiolytic, sedative, and analgesic properties of Dex might, at least partly, contribute to this long-lasting and accumulative analgesic effect (24 h) [54], [55]. These valid analgesic properties were also found in varied pain conditions, such as chronic constrictive injury (CCI) of the sciatic nerve-induced neuropathic pain [25], [56] and pH 5.0 PBS-induced acute inflammatory pain in mice [31], as well as bone cancer induced-pain in rats [57].

In our isobolographic analysis, an ED_50comb_ of 5.26 was less than ED_50add_ of 6.93 for Ropi, suggesting a synergistic analgesia. Importantly, reducing the LAs consumption means less neurotoxicity possibility to some extent. Thus, we highlighted that i.t. Dex as an adjuvant has obvious advantages over the individual medications. First, i.t. Dex seems to be an insufficient analgesia profile, while i.t. Ropi lacks long-lasting and accumulative analgesic effect. Second, a synergistic effect of i.t. Dex and Ropi might be beneficial in a long-term treatment because of more comprehensive effect of neuronal and astrocytic inhibition in the course of chronic pain. Third, smaller dosages of each agent may be used to reach equal or better efficacy. Finally, the LAs-induced side effects and motor function impacts would be minimized. More importantly, neurons might benefit from Dex’s protective effect. In this sense, the concomitant administration of Dex and Ropi may serve as a new analgesic strategy.

### Barrier between animal research and human application

Barrier lies between animal research and clinical application, and it does be a usual issue that a drug that works well in animals is ostensibly not effective in humans. One often-ignored explanation is the inappropriate translation of a drug dose from one animal species to another. The calculations for determining starting dose in humans as extrapolated from animals should use the more appropriate normalization of body surface area (BSA) than the body weight alone [58].

By using the following formula [59], we calculated the human equivalent dose (HED) based on our animal study: HED (mg/kg)  =  (mouse dose (mg/kg) * mouse K*_m_*)/human K*_m_* [59].

K*_m_* factor for rat and adult human being are 6 and 36, respectively.

The translated ED_50comb_ of i.t. Dex for a 70 kg adult is 36.59 μg, while the doses of i.t. Dex in clinical trials range largely from 3 to 15 μg [60]–[66]. Thus, we infer that the possible explanations for the discrepancy between animal studies and human trials might be that i) different stimulus intensity lies between surgical procedures or acute post-operative pain and chronic inflammatory pain; ii) higher concentration of LAs commonly applied in medical setting, which might achieve dominant in analgesia; iii) different interaction when i.t. Dex as an adjuvant combining with other specific LAs might contribute to diverse dose *vs* response relationship; iv) rats and human being demonstrates different sensitivity for adrenergic systems mediating by α_2_AR. However, these explanations need further experimental evidence.

## Conclusion

Our study shows that i.t. co-delivery of Dex and Ropi presents synergistic analgesia on the CFA-induced chronic inflammatory pain, in which spinal “neuron-astrocytic activation” mechanism may play an important role.

## Supporting Information

Figure S1 **Effect of repeated i.t. co-delivery of Dex and Ropi.**
(TIF)

Figure S2 **Effect of i.t. medications on motor function.**
(TIF)

Figure S3 **Effect of i.t. medications on pathology.**
(TIF)

File S1 **Supporting methods and results.**
(DOC)

## References

[1] Jones L, Othman M, Dowswell T, Alfirevic Z, Gates S, et al (2012) Pain management for women in labour: an overview of systematic reviews. Cochrane database of systematic reviews 3: CD009234. 10.1002/14651858.CD009234.pub2 PMC7132546 22419342

[2] Simmons SW, Taghizadeh N, Dennis AT, Hughes D, Cyna AM (2012) Combined spinal-epidural versus epidural analgesia in labour. Cochrane database of systematic reviews 10: CD003401. 23076897 10.1002/14651858.CD003401.pub3 PMC7154384

[3] Nishimori M, Low JH, Zheng H, Ballantyne JC (2012) Epidural pain relief versus systemic opioid-based pain relief for abdominal aortic surgery. Cochrane database of systematic reviews 7: CD005059. 10.1002/14651858.CD005059.pub3 22786494

[4] Gandhi K, Baratta JL, Heitz JW, Schwenk ES, Vaghari B, et al (2012) Acute pain management in the postanesthesia care unit. Anesthesiology clinics 30: e1–15. 23145460 10.1016/j.anclin.2012.09.001

[5] Dadure C, Capdevila X (2012) Peripheral catheter techniques. Paediatric anaesthesia 22: 93–101. 22050550 10.1111/j.1460-9592.2011.03730.x

[6] Goldman JA (1958) A rare toxic effect of local anaesthesia with lignocaine; a case report. British journal of anaesthesia 30: 377–379. 13572711 10.1093/bja/30.8.377

[7] Perrott DH (2008) Anesthesia outside the operating room in the office-based setting. Current opinion in anaesthesiology 21: 480–485. 18660657 10.1097/ACO.0b013e3283063499

[8] Harsten A, Kehlet H, Toksvig-Larsen S (2013) Recovery after total intravenous general anaesthesia or spinal anaesthesia for total knee arthroplasty: a randomized trial. British journal of anaesthesia. 10.1093/bja/aet104 23578860

[9] Zhong Z, Qulian G, Yuan Z, Wangyuan Z, Zhihua S (2009) Repeated intrathecal administration of ropivacaine causes neurotoxicity in rats. Anaesthesia and intensive care 37: 929–936. 20014599 10.1177/0310057X0903700612

[10] Sun ZH, Xu XP, Song ZB, Zhang Z, Wang N, et al (2012) Repeated intrathecal administration of ropivacaine causes neurotoxicity in rats. Anaesthesia and intensive care 40: 825–831. 22934865 10.1177/0310057X1204000427

[11] Sun Z, Liu H, Guo Q, Xu X, Zhang Z, et al (2012) In vivo and in vitro evidence of the neurotoxic effects of ropivacaine: the role of the Akt signaling pathway. Molecular medicine reports 6: 1455–1459. 23027315 10.3892/mmr.2012.1115

[12] Ihmsen H, Saari TI (2012) [Dexmedetomidine. Pharmacokinetics and pharmacodynamics]. Der Anaesthesist 61: 1059–1066. 23223843 10.1007/s00101-012-2114-1

[13] Stone LS, Broberger C, Vulchanova L, Wilcox GL, Hokfelt T, et al (1998) Differential distribution of alpha2A and alpha2C adrenergic receptor immunoreactivity in the rat spinal cord. The Journal of neuroscience : the official journal of the Society for Neuroscience 18: 5928–5937. 9671679 10.1523/JNEUROSCI.18-15-05928.1998 PMC6793037

[14] Mori K, Ozaki E, Zhang B, Yang L, Yokoyama A, et al (2002) Effects of norepinephrine on rat cultured microglial cells that express alpha1, alpha2, beta1 and beta2 adrenergic receptors. Neuropharmacology 43: 1026–1034. 12423672 10.1016/s0028-3908(02)00211-3

[15] Toda S, Sakai A, Ikeda Y, Sakamoto A, Suzuki H (2011) A local anesthetic, ropivacaine, suppresses activated microglia via a nerve growth factor-dependent mechanism and astrocytes via a nerve growth factor-independent mechanism in neuropathic pain. Molecular pain 7: 2. 21211063 10.1186/1744-8069-7-2 PMC3022746

[16] Chichorro JG, Lorenzetti BB, Zampronio AR (2004) Involvement of bradykinin, cytokines, sympathetic amines and prostaglandins in formalin-induced orofacial nociception in rats. British journal of pharmacology 141: 1175–1184. 15006904 10.1038/sj.bjp.0705724 PMC1574892

[17] Scholz J, Woolf CJ (2007) The neuropathic pain triad: neurons, immune cells and glia. Nature neuroscience 10: 1361–1368. 17965656 10.1038/nn1992

[18] Li X, Eisenach JC (2001) alpha2A-adrenoceptor stimulation reduces capsaicin-induced glutamate release from spinal cord synaptosomes. The Journal of pharmacology and experimental therapeutics 299: 939–944. 11714880

[19] Mei X, Wang W, Wang W, Li Y, Zhang H, et al (2009) Inhibiting astrocytic activation: a novel analgesic mechanism of ketamine at the spinal level? Journal of neurochemistry 109: 1691–1700. 19383087 10.1111/j.1471-4159.2009.06087.x

[20] Mei XP, Wang W, Wang W, Zhu C, Chen L, et al (2010) Combining ketamine with astrocytic inhibitor as a potential analgesic strategy for neuropathic pain ketamine, astrocytic inhibitor and pain. Molecular pain 6: 50. 20815929 10.1186/1744-8069-6-50 PMC2942826

[21] Mei XP, Zhang H, Wang W, Wei YY, Zhai MZ, et al (2011) Inhibition of spinal astrocytic c-Jun N-terminal kinase (JNK) activation correlates with the analgesic effects of ketamine in neuropathic pain. Journal of neuroinflammation 8: 6. 21255465 10.1186/1742-2094-8-6 PMC3033337

[22] Mei XP, Zhou Y, Wang W, Tang J, Wang W, et al (2011) Ketamine depresses toll-like receptor 3 signaling in spinal microglia in a rat model of neuropathic pain. Neuro-Signals 19: 44–53. 21389680 10.1159/000324293

[23] Wang W, Wang W, Mei X, Huang J, Wei Y, et al (2009) Crosstalk between spinal astrocytes and neurons in nerve injury-induced neuropathic pain. PloS one 4: e6973. 19759893 10.1371/journal.pone.0006973 PMC2736402

[24] Wang W, Wang W, Wang Y, Huang J, Wu S, et al (2008) Temporal changes of astrocyte activation and glutamate transporter-1 expression in the spinal cord after spinal nerve ligation-induced neuropathic pain. Anatomical record 291: 513–518. 10.1002/ar.20673 18384122

[25] Liu L, Ji F, Liang J, He H, Fu Y, et al (2012) Inhibition by dexmedetomidine of the activation of spinal dorsal horn glias and the intracellular ERK signaling pathway induced by nerve injury. Brain research 1427: 1–9. 22050961 10.1016/j.brainres.2011.08.019

[26] Xu B, Zhang WS, Yang JL, Lu N, Deng XM, et al (2010) Evidence for suppression of spinal glial activation by dexmedetomidine in a rat model of monoarthritis. Clinical and experimental pharmacology & physiology 37: e158–166. 20626414 10.1111/j.1440-1681.2010.05426.x

[27] Bourgoin S, Pohl M, Mauborgne A, Benoliel JJ, Collin E, et al (1993) Monoaminergic control of the release of calcitonin gene-related peptide- and substance P-like materials from rat spinal cord slices. Neuropharmacology 32: 633–640. 7689707 10.1016/0028-3908(93)90076-f

[28] Holz GGt, Kream RM, Spiegel A, Dunlap K (1989) G proteins couple alpha-adrenergic and GABAb receptors to inhibition of peptide secretion from peripheral sensory neurons. The Journal of neuroscience : the official journal of the Society for Neuroscience 9: 657–666. 2465394 10.1523/JNEUROSCI.09-02-00657.1989 PMC4516394

[29] Zimmermann M (1983) Ethical guidelines for investigations of experimental pain in conscious animals. Pain 16: 109–110. 6877845 10.1016/0304-3959(83)90201-4

[30] Kimura M, Saito S, Obata H (2012) Dexmedetomidine decreases hyperalgesia in neuropathic pain by increasing acetylcholine in the spinal cord. Neuroscience letters 529: 70–74. 22917606 10.1016/j.neulet.2012.08.008

[31] Zhang H, Zhou F, Li C, Kong M, Liu H, et al (2013) Molecular mechanisms underlying the analgesic property of intrathecal dexmedetomidine and its neurotoxicity evaluation: an in vivo and in vitro experimental study. PloS one 8: e55556. 23409000 10.1371/journal.pone.0055556 PMC3567091

[32] Guo BL, Sui BD, Wang XY, Wei YY, Huang J, et al (2013) Significant changes in mitochondrial distribution in different pain models of mice. Mitochondrion 13: 292–297. 23542162 10.1016/j.mito.2013.03.007

[33] Hargreaves K, Dubner R, Brown F, Flores C, Joris J (1988) A new and sensitive method for measuring thermal nociception in cutaneous hyperalgesia. Pain 32: 77–88. 3340425 10.1016/0304-3959(88)90026-7

[34] Zhuang ZY, Gerner P, Woolf CJ, Ji RR (2005) ERK is sequentially activated in neurons, microglia, and astrocytes by spinal nerve ligation and contributes to mechanical allodynia in this neuropathic pain model. Pain 114: 149–159. 15733640 10.1016/j.pain.2004.12.022

[35] Tallarida RJ (2001) Drug synergism: its detection and applications. The Journal of pharmacology and experimental therapeutics 298: 865–872. 11504778

[36] Sun YH, Dong YL, Wang YT, Zhao GL, Lu GJ, et al (2013) Synergistic Analgesia of Duloxetine and Celecoxib in the Mouse Formalin Test: A Combination Analysis. PloS one 8: e76603. 24116126 10.1371/journal.pone.0076603 PMC3792058

[37] Bai L, Wang W, Dong YL, Wang W, Huang J, et al (2012) Attenuation of mouse somatic and emotional inflammatory pain by hydralazine through scavenging acrolein and inhibiting neuronal activation. Pain physician 15: 311–326. 22828685

[38] Liang L, Tao B, Fan L, Yaster M, Zhang Y, et al (2013) mTOR and its downstream pathway are activated in the dorsal root ganglion and spinal cord after peripheral inflammation, but not after nerve injury. Brain research 1513: 17–25. 23583278 10.1016/j.brainres.2013.04.003 PMC3653996

[39] Yaster M, Guan X, Petralia RS, Rothstein JD, Lu W, et al (2011) Effect of inhibition of spinal cord glutamate transporters on inflammatory pain induced by formalin and complete Freund's adjuvant. Anesthesiology 114: 412–423. 21245732 10.1097/ALN.0b013e318205df50 PMC3057540

[40] Katsura H, Obata K, Miyoshi K, Kondo T, Yamanaka H, et al (2008) Transforming growth factor-activated kinase 1 induced in spinal astrocytes contributes to mechanical hypersensitivity after nerve injury. Glia 56: 723–733. 18293403 10.1002/glia.20648

[41] Zhuang ZY, Wen YR, Zhang DR, Borsello T, Bonny C, et al (2006) A peptide c-Jun N-terminal kinase (JNK) inhibitor blocks mechanical allodynia after spinal nerve ligation: respective roles of JNK activation in primary sensory neurons and spinal astrocytes for neuropathic pain development and maintenance. The Journal of neuroscience : the official journal of the Society for Neuroscience 26: 3551–3560. 16571763 10.1523/JNEUROSCI.5290-05.2006 PMC6673862

[42] Wirkner K, Gunther A, Weber M, Guzman SJ, Krause T, et al (2007) Modulation of NMDA receptor current in layer V pyramidal neurons of the rat prefrontal cortex by P2Y receptor activation. Cerebral cortex 17: 621–631. 16648456 10.1093/cercor/bhk012

[43] Jourdain P, Bergersen LH, Bhaukaurally K, Bezzi P, Santello M, et al (2007) Glutamate exocytosis from astrocytes controls synaptic strength. Nature neuroscience 10: 331–339. 17310248 10.1038/nn1849

[44] Oka M, Wada M, Wu Q, Yamamoto A, Fujita T (2006) Functional expression of metabotropic GABAB receptors in primary cultures of astrocytes from rat cerebral cortex. Biochemical and biophysical research communications 341: 874–881. 16455058 10.1016/j.bbrc.2006.01.039

[45] Thorlin T, Eriksson PS, Persson PA, Aberg ND, Hansson E, et al (1998) Delta-opioid receptors on astroglial cells in primary culture: mobilization of intracellular free calcium via a pertussis sensitive G protein. Neuropharmacology 37: 299–311. 9681928 10.1016/s0028-3908(98)00026-4

[46] Guo W, Wang H, Watanabe M, Shimizu K, Zou S, et al (2007) Glial-cytokine-neuronal interactions underlying the mechanisms of persistent pain. The Journal of neuroscience : the official journal of the Society for Neuroscience 27: 6006–6018. 17537972 10.1523/JNEUROSCI.0176-07.2007 PMC2676443

[47] Suter MR, Wen YR, Decosterd I, Ji RR (2007) Do glial cells control pain? Neuron glia biology 3: 255–268. 18504511 10.1017/S1740925X08000100 PMC2394739

[48] Watkins LR, Milligan ED, Maier SF (2001) Glial activation: a driving force for pathological pain. Trends in neurosciences 24: 450–455. 11476884 10.1016/s0166-2236(00)01854-3

[49] Werdehausen R, Fazeli S, Braun S, Hermanns H, Essmann F, et al (2009) Apoptosis induction by different local anaesthetics in a neuroblastoma cell line. British journal of anaesthesia 103: 711–718. 19700777 10.1093/bja/aep236

[50] Sites BD, Taenzer AH, Herrick MD, Gilloon C, Antonakakis J, et al (2012) Incidence of local anesthetic systemic toxicity and postoperative neurologic symptoms associated with 12,668 ultrasound-guided nerve blocks: an analysis from a prospective clinical registry. Regional anesthesia and pain medicine 37: 478–482. 22705953 10.1097/AAP.0b013e31825cb3d6

[51] Yan M, Dai H, Ding T, Dai A, Zhang F, et al (2011) Effects of dexmedetomidine on the release of glial cell line-derived neurotrophic factor from rat astrocyte cells. Neurochemistry international 58: 549–557. 21241763 10.1016/j.neuint.2011.01.013

[52] Bruzzone A, Pinero CP, Castillo LF, Sarappa MG, Rojas P, et al (2008) Alpha2-adrenoceptor action on cell proliferation and mammary tumour growth in mice. British journal of pharmacology 155: 494–504. 18604234 10.1038/bjp.2008.278 PMC2579667

[53] Sanders RD, Sun P, Patel S, Li M, Maze M, et al (2010) Dexmedetomidine provides cortical neuroprotection: impact on anaesthetic-induced neuroapoptosis in the rat developing brain. Acta anaesthesiologica Scandinavica 54: 710–716. 20003127 10.1111/j.1399-6576.2009.02177.x

[54] Grosu I, Lavand'homme P (2010) Use of dexmedetomidine for pain control. F1000 medicine reports 2: 90. 21283652 10.3410/M2-90 PMC3026617

[55] Huang R, Hertz L (2000) Receptor subtype and dose dependence of dexmedetomidine-induced accumulation of [14C]glutamine in astrocytes suggests glial involvement in its hypnotic-sedative and anesthetic-sparing effects. Brain research 873: 297–301. 10930558 10.1016/s0006-8993(00)02525-7

[56] Li SS, Zhang WS, Yang JL, Xiong YC, Zhang YQ, et al (2013) Involvement of protein kinase B/Akt in analgesic effect of dexmedetomidine on neuropathic pain. CNS neuroscience & therapeutics 19: 364–366. 23607700 10.1111/cns.12100 PMC6493625

[57] Yuan X, Wu J, Wang Q, Xu M (2013) The antinociceptive effect of systemic administration of a combination of low-dose tramadol and dexmedetomidine in a rat model of bone cancer pain. European journal of anaesthesiology. 10.1097/EJA.0b013e3283624a0f 23736095

[58] Reagan-Shaw S, Nihal M, Ahmad N (2008) Dose translation from animal to human studies revisited. FASEB journal : official publication of the Federation of American Societies for Experimental Biology 22: 659–661. 17942826 10.1096/fj.07-9574LSF

[59] Abbott FV, Grimes RW, Melzack R (1984) Single nerve capsaicin: effects on pain and morphine analgesia in the formalin and foot-flick tests. Brain research 295: 77–84. 6713178 10.1016/0006-8993(84)90817-5

[60] Gupta R, Verma R, Bogra J, Kohli M, Raman R, et al (2011) A Comparative study of intrathecal dexmedetomidine and fentanyl as adjuvants to Bupivacaine. Journal of anaesthesiology, clinical pharmacology 27: 339–343. 10.4103/0970-9185.83678 PMC3161458 21897504

[61] Gupta R, Bogra J, Verma R, Kohli M, Kushwaha JK, et al (2011) Dexmedetomidine as an intrathecal adjuvant for postoperative analgesia. Indian journal of anaesthesia 55: 347–351. 22013249 10.4103/0019-5049.84841 PMC3190507

[62] Solanki SL, Bharti N, Batra YK, Jain A, Kumar P, et al (2013) The analgesic effect of intrathecal dexmedetomidine or clonidine, with bupivacaine, in trauma patients undergoing lower limb surgery: a randomised, double-blind study. Anaesthesia and intensive care 41: 51–56. 23362890 10.1177/0310057X1304100110

[63] Mohamed AA, Fares KM, Mohamed SA (2012) Efficacy of intrathecally administered dexmedetomidine versus dexmedetomidine with fentanyl in patients undergoing major abdominal cancer surgery. Pain physician 15: 339–348. 22828688

[64] Shukla D, Verma A, Agarwal A, Pandey HD, Tyagi C (2011) Comparative study of intrathecal dexmedetomidine with intrathecal magnesium sulfate used as adjuvants to bupivacaine. Journal of anaesthesiology, clinical pharmacology 27: 495–499. 10.4103/0970-9185.86594 PMC3214555 22096283

[65] Al-Mustafa MM, Abu-Halaweh SA, Aloweidi AS, Murshidi MM, Ammari BA, et al (2009) Effect of dexmedetomidine added to spinal bupivacaine for urological procedures. Saudi medical journal 30: 365–370. 19271064

[66] Kanazi GE, Aouad MT, Jabbour-Khoury SI, Al Jazzar MD, Alameddine MM, et al (2006) Effect of low-dose dexmedetomidine or clonidine on the characteristics of bupivacaine spinal block. Acta anaesthesiologica Scandinavica 50: 222–227. 16430546 10.1111/j.1399-6576.2006.00919.x

