# Supplementary figures and images for: Inhibiting Spinal Neuron-Astrocytic Activation Correlates with Synergistic Analgesia of Dexmedetomidine and Ropivacaine
Source: PLoS One. 2014 Mar 21;9(3):e92374. doi: 10.1371/journal.pone.0092374 (PMC3962412; doi:10.1371/journal.pone.0092374)

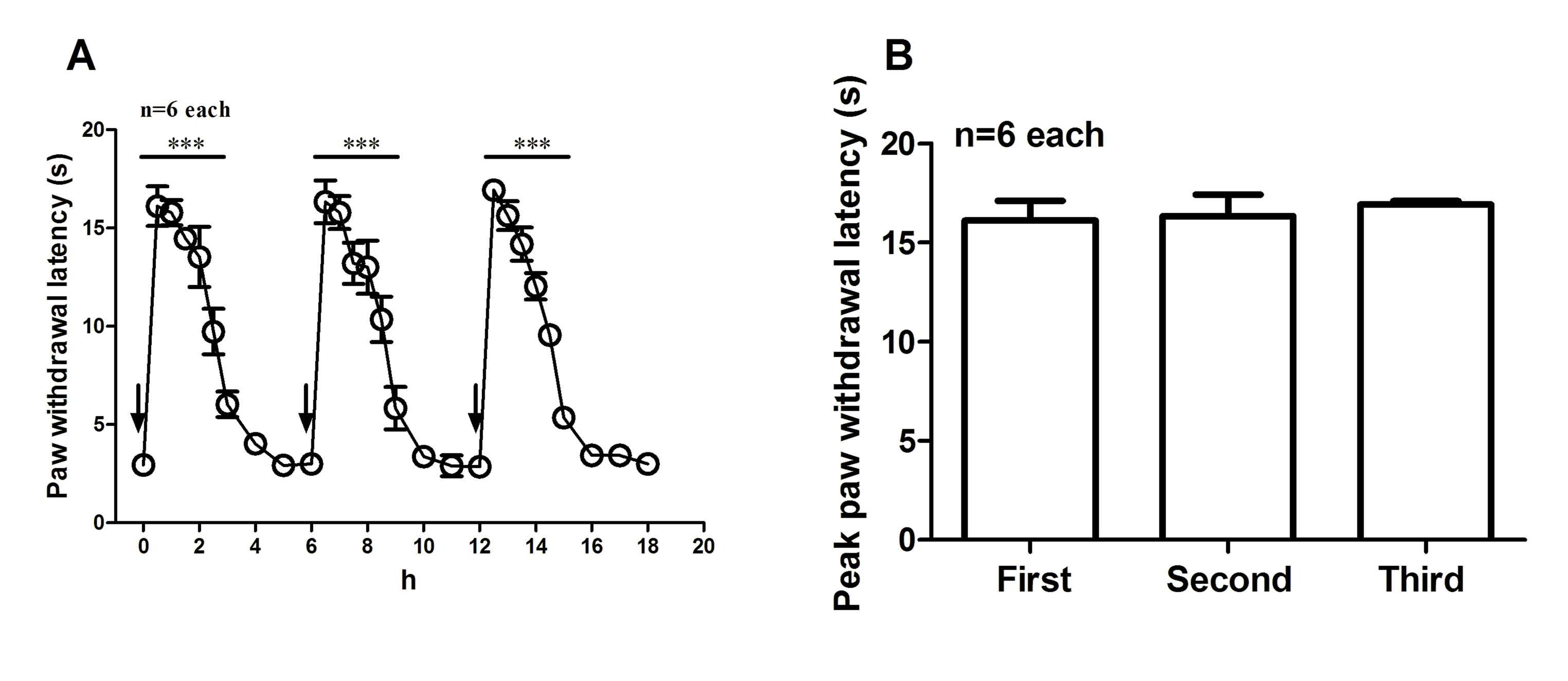

Supplement: Figure S1 — Effect of repeated i.t. co-delivery of Dex and Ropi. (TIF) [file pone.0092374.s001.tif]

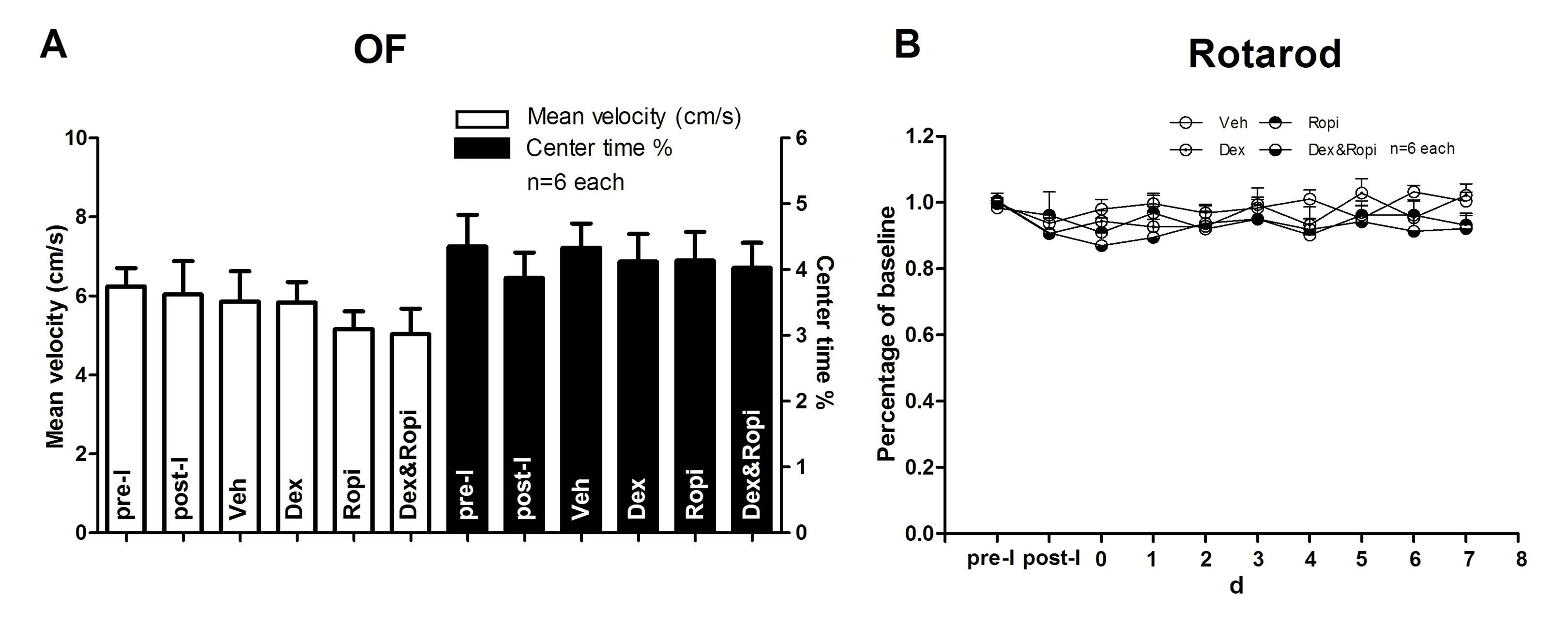

Supplement: Figure S2 — Effect of i.t. medications on motor function. (TIF) [file pone.0092374.s002.tif]

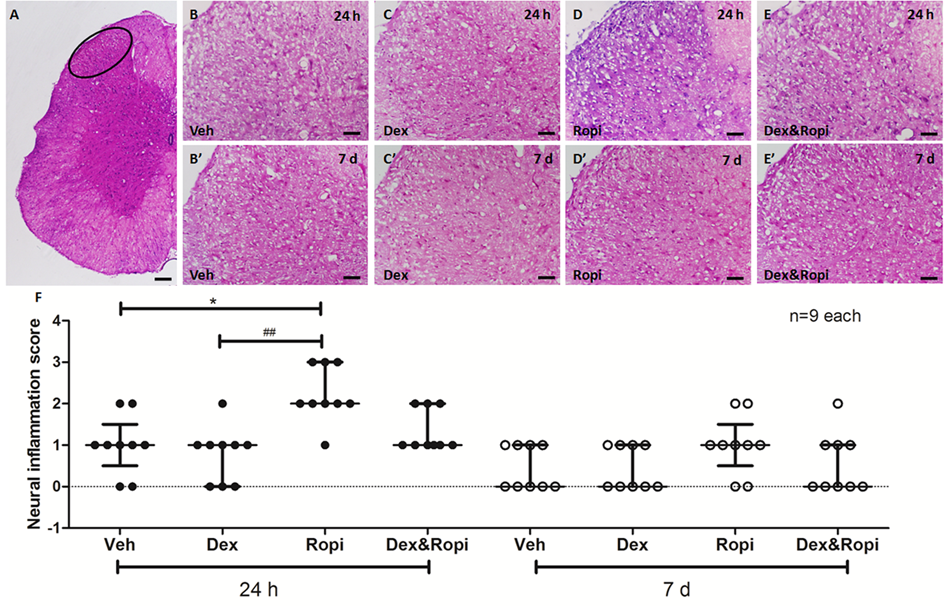

Supplement: Figure S3 — Effect of i.t. medications on pathology. (TIF) [file pone.0092374.s003.tif]
