# Supplementary material for: Inhibiting Spinal Neuron-Astrocytic Activation Correlates with Synergistic Analgesia of Dexmedetomidine and Ropivacaine
Source: PLoS One. 2014 Mar 21;9(3):e92374. doi: 10.1371/journal.pone.0092374 (PMC3962412; doi:10.1371/journal.pone.0092374)
Supplement: File S1 — Supporting methods and results. (DOC) [file pone.0092374.s004.doc]

**SUPPORTING INFORMATION**

**Experimental protocols**

Supplemental experiemnt was designed to evaluate the potential tachyphylaxis or sensitization effect when i.t. delivery of ED_50comb_ Dex&Ropi was repeated for 3 times at a 6-h interval after s.c. CFA injection. PWLs were measured at 30 min or 60 min interval during the first or second 3 h following each i.t. injection, respectively, to evaluate the analgesic duration and intensity for i.t. delivery of ED_50comb_ Dex&Ropi.

For an ideal analgesic strategy, fewer side effects are always expected. Considering that the effects of intubation procedure and i.t. drug delivery used in the current experiments might influence motor function and bias our behavioral readout, we further performed open field (OF) and rotarod tests on naïve rats free from CFA injection or pain behavioral observation after maximal dosages of drugs (or their combination) were administered. In this section, the OF test was done at 1 d pre- and 3 d post-intubation, as well as at 30 min after i.t. injection with vehicle or drugs. The rotarod test was done at 1 d pre-, 3 d post-intubation, and 30 min after daily i.t. injection with vehicle or drugs until day 7.

**Behavior test**

**OF test** The OF test was performed according to our previous study . Rats were placed at the center of a cubic chamber [100 cm (W) × 100 cm (H) × 50 cm (D)]. The locomotion of rats traveled in 15 min was monitored with an automated analyzing system (Shanghai Mobiledatum Information Technology Co., Ltd). The mean velocity was used as a parameter for the locomotion and the percentage of time spent in the center area (center time %) is a parameter to evaluate anxiety/depression levels by off-line analysis. All animals were habituated to the testing room for 30 min before the start of observation. The test room was dimly illuminated with indirect white lighting, as rats were nocturnal and their natural exploratory behavior was hindered in well-illuminated conditions.

**Rotarod test** According to our previous studies , a standard rat rotarod test was used to determine the rats’ motor coordination and balance. Rats naïve to the rotarod test were placed on the Ugo Basile 7650 Rotarod accelerator treadmill (Ugo Basile, Varese, Italy) set at the minimal speed for training sessions of 1-2 min at an interval of 30 min. After this adaption period, the animals were placed on to the rotarod at a constant speed of 25 rpm. As the animal took a grip on the drum, the accelerator mode was selected on the treadmill, i.e. the rotation rate of the drum was increased linearly at 20 rpm. Thereafter, the time was measured from the start of the acceleration period until the rat fell down the drum. The cut-off time was 30 s. The time that the animal remained on the rotarod was recorded and expressed as a percentage of its own baseline value.

**Pathological HE staining and inflammation scoring** Rats were perfused and the spinal cords were removed as described above in the immunofluorescence histochemical staining methods. Transverse frozen spinal sections (10 μm thick) were cut and mounted on slides until dry to process for HE staining by using routine techniques as described in previous studies to detect the general morphological changes. Briefly, these dried slices were immersed in distilled water for 1 min, and then stained with hematoxylin (Roth, Karlsruhe, Germany) for 10 min and eosin (Merck, Darmstadt, Germany) for 30 s, respectively. Between each staining, the slices were differentiated with acid alcohol and checked staining after blueing-up followed by washing up with distilled water, and then dehydrated through gradient ethanol solution from 70% to 100% and immersed in dimethylbenzene. Finally, the stained sections were covered with polystyrene and placed under a cover glass.

Inflammation score was quantitatively analyzed according to previous studies . Inflammation score = 0: The neural space is void of any significant inflammatory cells. Inflammation score = 1: Focal portions of inflammatory cells infiltration involving 5-10% of the sections. Inflammation score = 2: Moderate degree of inflammatory cells infiltration. Inflammation score = 3: Severe inflammation is seen with large number of inflammatory cells infiltration in the sections.

**RESULTS**

**Effect of repeated i.t. co-delivery of Dex and Ropi**

Our isobolographic analysis demonstrated that i.t. Dex and Ropi combination presented a synergistic effect in a short-term observation, however, whether any acute tachyphylaxis or sensitization would occur when the combination was repeatedly administrated to achieve a long-term analgesia remained unclear. Thus, we conducted the following experiment to solve this issue.

To investigate the effect of repeated i.t. Dex and Ropi combination, rats received thrice i.t. ED_50comb_ Dex&Ropi at a 6 h interval. During 6 h after each injection, thermal pain behaviors were continuously monitored. The results revealed that each injection presented a similar analgesic time-course and intensity (**Figure S1A** and **B**; one-way ANOVA, *P* = 0.7898). These data suggested that repeated i.t. Dex and Ropi combination presented a stable analgesia property without any acute tachyphylaxis or sensitization in treating chronic inflammatory pain.

**Effect of i.t. medications on motor function**

There was no significant group difference in the locomotion revealed by the mean velocity during the 15 min recording time in OF test (**Figure S2A**; one-way ANOVA, *P* = 0.7009). *Bonferroni’s post hoc* test also revealed no significant group difference among intubation procedure, i.t. Dex 2 μg/200 g (*P* > 0.05), Ropi 20 μg/200 g (*P* > 0.05), or Dex 1.3208 μg/200 g and Ropi 11.08 μg/200 g combination (*P* > 0.05) and vehicle groups. In the OF test, there was no significant group difference in the percentage of center time (**Figure S2A**; one-way ANOVA, *P* = 0.9610), indicating no difference in their anxiety/depression status.

Results from rotarod test showed that neither intubation procedure, i.t. Dex 2 μg/200 g, Ropi 20 μg/200 g, nor Dex 1.3208 μg/200 g and Ropi 11.08 μg/200 g combination affected the motor performance in rats (**Figure S2B**; two-way ANOVA, *p* > 0.05). It assured that the anti-hyperalgesia or anti-nociceptive effects of i.t. medications were not derived from the impairments of motor function.

**Effect of i.t. medications on pathology**

Since potential neuroinflammation and neurotoxicity might restrict the i.t. adjuvant’s application, neuroprotective effect is highlighted for an ideal analgesic strategy. Thus, we conducted the following experiment to explore the potential lesions of i.t. drugs on the SDH. HE staining of ipsilateral SDH was performed at 24 h and on 7 d after s.c. CFA injection. SDH in the Ropi group had higher inflammation scores at 24 h when compared with the vehicle control (*Kruskal-Wallis* with *Dunn's* multiple comparison test, *P* < 0.05). i.t. Dex tended to attenuate Ropi-induced actue neuroinflammation at 24 h after CFA injections. Similar inflammation scores among Veh, Dex, and Dex&Ropi group at 24 h were observed (*Kruskal-Wallis* with *Dunn's* multiple comparison test, *P* > 0.05). On day 7, SDH in all groups were completely normal with inflammation scores (*Kruskal-Wallis* test, *P =* 0.2566) (**Figure S3**).

**REFERENCES**

1 Woolf CJ, American College of P and American Physiological S (2004) Pain: moving from symptom control toward mechanism-specific pharmacologic management. Annals of internal medicine 140: 441-451.

2 Rondon LJ, Privat AM, Daulhac L, Davin N, Mazur A, et al. (2010) Magnesium attenuates chronic hypersensitivity and spinal cord NMDA receptor phosphorylation in a rat model of diabetic neuropathic pain. The Journal of physiology 588: 4205-4215.

3 Willis WD (2002) Long-term potentiation in spinothalamic neurons. Brain research Brain research reviews 40: 202-214.

4 Mao J, Price DD, Phillips LL, Lu J and Mayer DJ (1995) Increases in protein kinase C gamma immunoreactivity in the spinal cord dorsal horn of rats with painful mononeuropathy. Neuroscience letters 198: 75-78.

5 Li LY, Li JL, Zhang HM, Yang WM, Wang K, et al. (2013) TGFbeta1 treatment reduces hippocampal damage, spontaneous recurrent seizures, and learning memory deficits in pilocarpine-treated rats. Journal of molecular neuroscience : MN 50: 109-123.

6 Brummett CM, Norat MA, Palmisano JM and Lydic R (2008) Perineural administration of dexmedetomidine in combination with bupivacaine enhances sensory and motor blockade in sciatic nerve block without inducing neurotoxicity in rat. Anesthesiology 109: 502-511.

7 Toumpoulis IK, Papakostas JC, Matsagas MI, Malamou-Mitsi VD, Pappa LS, et al. (2004) Superiority of early relative to late ischemic preconditioning in spinal cord protection after descending thoracic aortic occlusion. The Journal of thoracic and cardiovascular surgery 128: 724-730.

**Supporting Information Legends**

**Figure S1.** **Effect of repeated i.t. co-delivery of Dex and Ropi.** Repeatedly i.t. concomitant administration of ED_50comb_ Dex&Ropi presented similar analgesia profile both in analgesia duration (**A**: *** *p*<0.001, compared with 0 min time point; arrows indicated i.t. intervention time point) and intensity (**B**).

**Figure S2.** **Effect of i.t. medications on motor function.** i.t. intubation (post-I), 2 μg/200 g Dex, 20 μg/200 g Ropi or their ED_50add*8/10_ combination (ED_50add*8/10_ Dex&Ropi) did not affect animals’ locomotion and motor coordination. Mean velocity and center time% in OF (**A**), as well as latency to fall in rotarod test (**B**) were used to detect the locomotion and motor coordination, respectively.

**Figure S3.** **Effect of i.t. medications on pathology.** Effects of each i.t. intervention on spinal neuroinflammation at 24 h and on 7 d were shown in **B~E** and **B’~E’**, respectively. Scheme showed an overview of detected region (**A**). i.t. Dex tended to attenuate Ropi-induced actue neuroinflammation at 24 h after s.c. CFA injection (**B~E**). No significant pathological injuries were observed on 7 d (**B’~E’**). Neural inflammation score for each intervention group was presented in **F**.

* *p*<0.05, compared with Veh group; ## *p*<0.01, compared with Dex group. Scalebars=100 μm in **A**, 50 μm in **B~E** and **B’~E’**.
